# Supplementary material for: Effects of rumen-protected methionine supplementation on the performance of high production dairy cows in the tropics
Source: PLoS One. 2021 Apr 30;16(4):e0243953. doi: 10.1371/journal.pone.0243953 (PMC8087032; doi:10.1371/journal.pone.0243953)
Supplement: S1 Table — (PDF) [file pone.0243953.s001.pdf]

Effects of the dietary treatments on milk yield and composition in dairy cows.

	Diet[1]			*P*-value		
**Item**	**CON**	**SM**	SEM	Diet	week	Diet x week
Milk yield, kg/d	40.0	41.7	0.50	0.03	0.19	0.87
ECM[2], kg/d	38.0	41.0	0.67	<0.01	0.07	0.99
FCM[3], 3.5%	38.3	41.1	0.77	0.01	0.14	0.97
Milk fat, %	3.21	3.41	0.07	0.06	0.85	0.99
Milk fat, kg/d	1.29	1.42	0.04	0.02	0.64	0.99
Milk protein, %	2.97	3.14	0.03	<0.01	<0.01	0.95
Milk protein, kg/d	1.19	1.30	0.01	<0.01	<0.01	0.95
Milk casein, %	2.28	2.39	0.03	<0.01	<0.01	0.96
Milk lactose, %	4.61	4.54	0.02	0.03	<0.01	0.83
Milk lactose, kg/d	1.85	1.89	0.03	0.26	0.02	0.80
Total solids, %	11.78	12.10	0.13	0.10	0.42	0.96
Total solids, kg/d	4.75	5.01	0.09	0.05	0.20	0.96
MUN, mg/dL	10.43	10.74	0.24	0.37	<0.01	<0.01
SCC, X10^3	135.86	180.50	44.70	0.48	0.13	0.37

[1]CON = Control diet; SM = CON + SM (0.09 % of DMI)

[2]Energy-corrected milk (kg/d) = [(0.323 x kg milk) + (12.82 x kg fat) + (7.13 x kg true protein)] (Hutjens, 2010).

[3]Fat-corrected milk (3.5%) = 0.4318 kg milk + 16.23 kg milk fat
