# Supplementary material for: Effects of rumen-protected methionine supplementation on the performance of high production dairy cows in the tropics
Source: PLoS One. 2021 Apr 30;16(4):e0243953. doi: 10.1371/journal.pone.0243953 (PMC8087032; doi:10.1371/journal.pone.0243953)
Supplement: S2 Table — (PDF) [file pone.0243953.s002.pdf]

Effect of dietary RPMet supplementation on plasma AA concentrations (μM) of dairy cows[1].

	Diet[2]		
	CON	SM	*P*-value
Arg	77.1 ± 4.8	86.0 ± 6.8	0.29
His	33.5 ± 3.8	31.7 ± 5.3	0.79
Ile	78.7 ± 5.2	70.0 ± 7.3	0.34
Leu	73.7 ± 7.5	70.0 ± 10.5	0.78
Lys	57.4 ± 4.5	64.7 ± 6.3	0.36
Met	18.4 ± 1.4	29.6 ± 1.9	**<0.001**
Phe	38.7 ± 1.6	39.0 ± 2.3	0.91
Thr	54.0 ± 3.6	62.1 ± 5.0	0.21
Trp	31.2 ± 1.7	32.3 ± 2.4	0.73
Val	220 ± 16.4	212 ± 22.9	0.76
Ala	206 ± 15.9	272 ± 22.3	**0.03**
Asn	24.0 ± 2.2	30.5 ± 3.1	0.10
Gln	95.4 ± 8.3	90.6 ± 11.6	0.74
Glu	63.4 ± 4.3	59.6 ± 6.0	0.61
Pro	72.9 ± 5.4	84.4 ± 7.5	0.23
Ser	104 ± 15.8	97.7 ± 22.2	0.80
Tyr	38.7 ± 4.4	39.1 ± 6.1	0.95

[1]Data are presented as mean ± SEM; [2] CON = Control diet; SM = CON + SM (0.09 % of DMI)
