# Supplementary figures and images for: Effects of rumen-protected methionine supplementation on the performance of high production dairy cows in the tropics
Source: PLoS One. 2021 Apr 30;16(4):e0243953. doi: 10.1371/journal.pone.0243953 (PMC8087032; doi:10.1371/journal.pone.0243953)

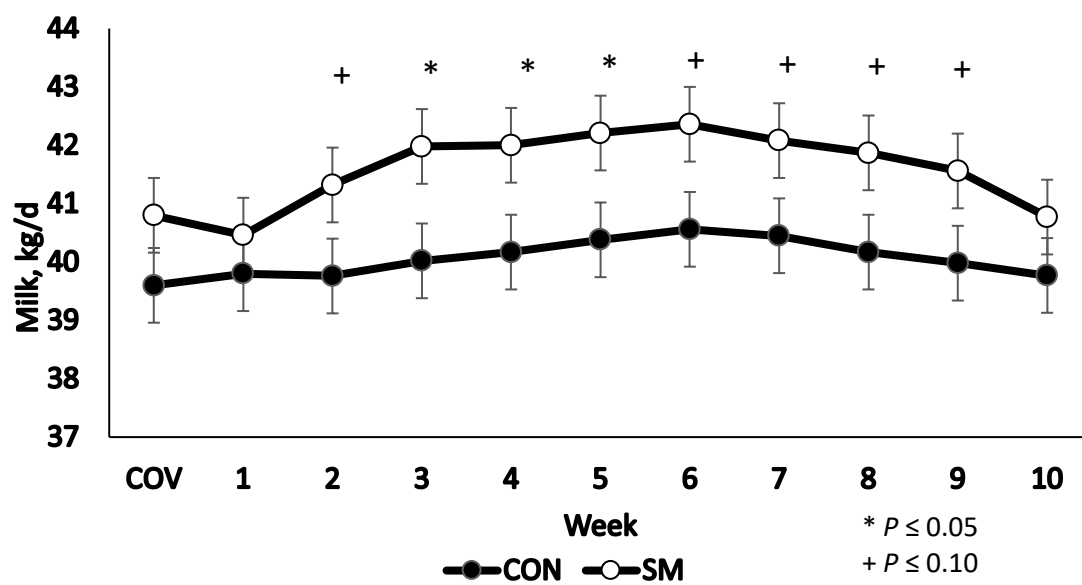

Supplement: S1 Fig — (PDF) [file pone.0243953.s003.pdf]
