# Supplementary material for: Effects of rumen-protected methionine supplementation on the performance of high production dairy cows in the tropics
Source: PLoS One. 2021 Apr 30;16(4):e0243953. doi: 10.1371/journal.pone.0243953 (PMC8087032; doi:10.1371/journal.pone.0243953)
Supplement: Attachment — Submitted filename: Response to reviewers.docx [file pone.0243953.s004.docx]

**Response to reviewers:**

L15: What do you mean with adequate? It is not a correct form to state it.

**“…. containing adequate protein and energy levels” revised to “with protein and energy levels calculated according to the literature”**

L19: Please put the name of the methionine group in the brackets (I assume SM).

**Revised in the manuscript according to the suggestion of the reviewer**

L21: If the experiment lasts 10 wk, why did authors collect blood samples at 30 d? BCS and BW was measured at the end though.

**We started the collects at the beginning of the experiment but found that the methodology used for plasma AA used here in Brazil had a mistake. Therefore, we decided to sample again at 30 d for sending the samples to a USA lab. Unfortunately, took more than four months to get the authorization for the carriage of biological samples into USA.**

**We decided that at 30 days of trial, the cows were already adapted to their diets and could reflect the variation on blood amino acid profile.**

L23-27: For some variables, authors state the exact p value and for others they indicate with ≤. Please be consistent. In addition, I assume that authors have only a p value (CON vs SM, overall) for ECM, milk protein yield, milk protein %, and casein, so why do not they use the exact p value?

**Revised in the manuscript according to the suggestion of the reviewer**

L73-81: Not only for the mechanisms pointed out, but also for the antioxidant system (taurine and glutathione). Please re-phrase this part taking into account these functions linked to the methionine cycle and transsulfuration pathway.

**Revised to: In addition to SAM, methionine acts as precursor of hydrogen sulfide, taurine, and glutathione. These products are reported to alleviate oxidant stress induced by various oxidants and protect the tissue from the damage**

L81: Please, add the reference.

**Reference added**

L87: In the authors’ hypothesis should be also stated the potential changes in the plasma AA profile.

**Revised to: “…and can affect the plasma AA profile”**

L91: It is not correct “early lactation cows”. Authors mentioned the first half and since they started on average at 65 DIM and concluded 10 wk after, it cannot be considered early lactation. Re-phrase according to this comment.

**Revised to mid-lactation**

L92: “correct amount” to reach the ratio required for the experiment objective. There is not an absolute right amount of Met supplementation.

**Revised to “by supplying RPMet to a basal diet formulated for high milk production.”**

L102-105: Since authors stressed out the tropical environment in the hypothesis/objective, it would be good to add some info on the climatic condition during the trial. It seems important to have these data, even though there is not a comparison with other climes such as North America and Europe.

**Local THI information during the experiment was included**

L102: equipped is not the right word.

**Revised to “two separate single pens, one for each treatment, in a free-stall barn with sand bedding and a cooling system…”**

L104: Please make clearer this point.

**Revised to “A total mixed ration (TMR) was offered twice daily (at 06:00 and 15:00 h). Feed allowance was adjusted daily to ensure 5% feed refusals.”**

L107-114: Please put the average of milk yield, bw, bcs and DIM at the beginning of the experiment in the table with the two groups separated.

**Averages included in Table 1 according the recommendation**

L118-122: Authors have already these data in table, thus it does not need to be reported again.

**Written values were excluded from the paragraph**

L123-124: It is not clear how authors obtained the 14 g/d of true Met starting from 23 g/d of Smartamine. Did the authors conducted in vitro/in vivo experiment to establish the true by-pass Met? If not, it is better to report the Samrtamine addition as % of DM and then specifying the % of Met provided by Smartamine, as authors stated in the L125.

**Revised to “SMARTAMINE^®^ M is a lipid/pH-sensitive polymer-protected product that contains 75% DL-Met with an assumed Met bioavailability value of 80% [9]. From those values, the Met-supplemented diet (SM) consisted of the basal diet plus 23 g/d of SMARTAMINE^®^ M (Adisseo Inc., Antony, France) to provide 14 g/d of additional metabolizable Met.”**

Table 1: I guess there is a mistake indicating “0.09% of DMI”. Probably authors were supposed to write “0.09% of DM”. If authors specify DMI lead to a mistake because it assumes that the 0.09% refers to the true DMI. Or referring as 0.09% of predicted DMI. Up to authors.

**Revised to 0.09% of DM**

Was the Met provided as top-dress or mixed in the TMR?

**Mixed in the TMR included in L133**

Table 2: The authors could even eliminate the table since it does not give relevant support at the paper. Those values come up from prediction equations, and authors do not have DMI data.

**We would like to maintain the Table 2 because think that the predicted flows can help to understand the flux increase of the supplement methionine and is necessary to decide how much Smartamine M to feed. We revised the text before the Tables to**

**“The nutritional composition of the experimental diets and predicted protein balances are shown in Table 1, calculated according to the NRC (2001). Predicted flows of EAA to the small intestine (NRC, 2001) are shown in Table 2. Model-predicted flows of MP and EAA indicated that 14 g/d of additional metabolizable Met was needed to achieve an approximate predicted Lys/Met ratio of 3.00/1 in metabolizable protein, With a minimum content of 75% DL-Met and an assumed Met bioavailability value of 80%, this required 23/d of SMARTAMINE^®^ M.”**

L163: Milk samples were collected from one milking or composited from the 3 milking of the same day?

**Revised to “Composite milk samples were made for each cow from the 3 milkings of the same day once during the adaptation period and every two weeks until the end of the study.”**

L175: 12 cows for blood samples up to 38 cows totally involved in each group seems a little number of subjects. Could authors explain why?

**Based on Toledo et al., 2017, 10% of cows from each treatment is enough to show the difference on plasm AA level after 12 hours of MET supplementation. They used 20, 40 and 65 individual samples from an experimental herd of 309 lactating cows. In our study, according to the power calculation of SAS, a sample of 30% of 76 cows should be enough to find differences between treatments.**

Why only at 30 days after starting treatment and not at the end or both?

**At 30 days of trial, the cows were already adapted to their diets and could reflect the variation on blood amino acid profile.**

L177: Why 6 hours after delivering the first TMR?

**Revised to “Blood was collected from the tail vein after the second milking, about 6 hours after the 1^st^ feeding..”**

**Since the RPMet is mixed in the TMR and the feed intake is continuous, the AA plasma levels are almost constant. The 6 hours period after the first meal was a consequence of the 2^nd^ milking occasion.**

L236: Individual DMI? I guess not, so I suggest stating clearly that is an average of the group-fed cows in each treatment. According to this, I suggest also not to speculate too much.

**Revised to “..the DMI average of the group-fed cows in each treatment was not affected (*P*>0.05) by treatment (26.2 kg/d for CON vs. 26.1 kg/d for SM).”**

L281: Very interesting result but I would like to see data during the 2-wk of adaptation according to the treatment (SM and CON) and if the covariate inclusion was significant in the model.

**The covariate period was included in the Fig 1 according the Reviewer recommendation.**

L308-313: How can it be attached to the previous paragraph discussing the milk lactose? It is in contrast, firstly, and I do not see the link between these two aspects considering also the lack of data on blood glucose concentration.
However, it is not correct talking about inflammatory state because authors do not have data on blood biomarkers. I would eliminate this part.

**Paragraph eliminated according the Reviewer recommendation.**

L349-353: Unfortunately, authors do not have data to discuss about this. Consider eliminating and I suggest the authors to keep the discussion according to their data.

**Eliminated according the Reviewer recommendation.**

Reviewer #2: This is a well written manuscript with concise hypothesis and objectives. The results seem to be a straightforward interpretation and, for the most part, are in line with prior data on this topic. The main concern of this reviewer is the unclear collection, data analysis, and interpretation of individual or collective (e.g., pen) DMI. This needs to be clarified before this manuscript can be suitable for publication.

Lines 104-105 “A total mixed ration (TMR) was offered…”

**OK**

Line105 Explain how this was calculated. Was this done by total TMR offered to the pen? Were the 38 cows per treatment kept on single separate pens, or were there multiple pens?

**Revised to “Cows were housed in two separate single pens, one for each treatment, in a free-stall barn with sand bedding and a cooling system…”**

Lines 109-110 What about previous lactation yield or 305ME corrected lactation yield?

**Revised to “Seventy-six lactating multiparous Holstein cows with a previous lactation average milk yield of 10600 kg..”**

Line 117 Please correct this “(indicate what you used)”.

**OK**

Lines 119-122 Please reference Table 1 here.

**Written values were excluded from the paragraph according recommendation of the Reviewer 1 “**L118-122: Authors have already these data in table, thus it does not need to be reported again.”

Line 146 “Predicted DM intake” instead of “average DM intake”. Also, what about milk yield, milk composition, BW, and BCS? All these parameters are required for NRC to make predictions on requirements for RDP, RUP, and MP.

**Revised to ^“^Predicted using NRC (2001)” and removed “average DM intake of 26.3 kg/d”**

Line 198 Delete “and cow”

**OK**

Line 219 “RPMet”

**OK**

Lines 236-237 Where are these data coming from? From the Materials and Methods section, it is not clear how the authors collected individual DMI.

**Revised to “…the DMI average of the group-fed cows in each treatment was not affected (*P*>0.05) by treatment (26.2 kg/d for CON vs. 26.1 kg/d for SM).”**

**Also included in Materials and Methods: “Feed allowance and refusal amounts were recorded daily to calculate the group feed intakes.”**

Line 270 Provide references.

**OK**

Line 273 Which AA? The authors should better this theory and Lines 277-278 How does this relate to a more efficient use of other absorbed AA?
**Revised to: …we suggest that the milk yield response in this experiment was due to increased efficiency of use of the other absorbed proteinogenic AA by the mammary gland for milk component synthesis. There are three reasons for this suggestion. The first is that the predicted supply of ME for the achieved milk yield was not limiting, hence there was an opportunity for increased milk yield without increased feed intake. The second factor is that milk yield is a function of milk component synthesis, particularly lactose and protein. It is well established that yields of lactose and protein are highly and positively correlated with milk yield. And third, in addition to being a substrate limiting AA for protein synthesis, it also possesses signaling properties, along with leucine, isoleucine and histidine, for promoting cellular anabolic metabolism. It has been shown to activate protein translation by being mechanistically linked to mTOR regulation (Sabatini et al., 2017). The result, if this occurred in our study, would be increased efficiency of use of all AA for protein synthesis.**

Lines 305-306 Please clarify, do the authors meant that the mammary gland has the flexibility to use other substrates for gluconeogenesis (e.g., BHB and lactate)? If so, provide references.

**Revised to: “Doepel and Lapierre [33] observed a positive response of the mammary gland of dairy cows to EAA infusion, increasing milk and milk protein yields, without a concurrent increase in glucose uptake but with an increase of the uptake of BHBA plus lactate, and concluded that this intensification of use of other energy-yielding substrates, demonstrates the metabolic priority of milk production and the flexibility of the mammary gland to use various substrates at its disposal to support higher milk and protein yields.”**

Line 307 Not clear what is “it”?

**Revised to “…to support higher milk and protein yields.”**

Line 318 Not clear what the authors meant by “fractional protein synthesis”.

**Removed**

Lines 318-319 Provide reference.

**OK**
Lines 319-321 Provide reference.

**OK**

Line 324 “maximum protein yield”

**OK**
Lines 353-355 Provide reference.

**OK**

Line 368 “(P< 0.05)”

**Revised**

Line 401 This reviewer suggests changing this to “Met was a limiting nutrient…” Since this experiment was not designed to compare Met against other nutrients, this statement needs to be reworded to be in agreement with the experimental design and results.

**Revised to “A positive response in milk yield and milk component was observed by supplying RPMet to a basal diet for mid-lactation dairy cows. We conclude that Met was limiting in this experiment and formulating rations for a more ideal Lys/Met ratio of absorbed AA was of significant benefit to the cows.”**

Line 406 This reviewer suggest to double-check these uM units here and in Table 4. This unit seem to be “uM” instead of “uM/mL”.

**OK**
